# Live-cell imaging of single mRNA dynamics using split superfolder green fluorescent proteins with minimal background

**DOI:** 10.1261/rna.067835.118

**Published:** 2020-01

**Authors:** Sung Young Park, Hyungseok C. Moon, Hye Yoon Park

**Affiliations:** 1 Department of Physics and Astronomy, Seoul National University, Seoul, 08826, Korea; 2 Center for RNA Research, Institute for Basic Science, Seoul, 08826, Korea; 3 Institute of Applied Physics, Seoul National University, Seoul, 08826, Korea; 4 Institute of Molecular Biology and Genetics, Seoul National University, Seoul, 08826, Korea

**Keywords:** single-molecule imaging, live-cell imaging, MS2 system, fluorescence complementation

## Abstract

The MS2 system, with an MS2 binding site (MBS) and an MS2 coat protein fused to a fluorescent protein (MCP–FP), has been widely used to fluorescently label mRNA in live cells. However, one of its limitations is the constant background fluorescence signal generated from free MCP–FPs. To overcome this obstacle, we used a superfolder GFP (sfGFP) split into two or three nonfluorescent fragments that reassemble and emit fluorescence only when bound to the target mRNA. Using the high-affinity interactions of bacteriophage coat proteins with their corresponding RNA binding motifs, we showed that the nonfluorescent sfGFP fragments were successfully brought close to each other to reconstitute a complete sfGFP. Furthermore, real-time mRNA dynamics inside the nucleus as well as the cytoplasm were observed by using the split sfGFPs with the MS2–PP7 hybrid system. Our results demonstrate that the split sfGFP systems are useful tools for background-free imaging of mRNA with high spatiotemporal resolution.

## INTRODUCTION

Fluorescent proteins (FPs) fused to RNA binding proteins (RBPs) have been widely used for visualizing the transport and dynamics of RNA in living cells (Tyagi 2009; Rath and Rentmeister 2015). In particular, the MS2 system using the high affinity interaction between the MS2 coat protein (MCP) and the MS2 binding site (MBS) (Bertrand et al. 1998; Beach et al. 1999) has been extensively adopted for tagging a specific RNA, enabling the study of RNA localization and dynamics in many different organisms, from bacteria to mammalian cells and tissues (Bertrand et al. 1998; Rook et al. 2000; Forrest and Gavis 2003; Fusco et al. 2003; Golding and Cox 2004; Chubb et al. 2006; Lionnet et al. 2011; Park et al. 2014). To distinguish the RNA tagged with the MCP fused with a fluorescent protein (MCP–FP) from the constant background of unbound MCP–FPs, two strategies have been utilized: (i) introducing multiple repeats of the MBS motif into the target RNA, and (ii) attaching a nuclear localization sequence (NLS) to the MCP–FP to accumulate the unbound MCP–FPs in the nucleus to reduce the background in the cytoplasm (Fusco et al. 2003). By applying this approach, one can label a target RNA with multiple FPs and obtain a high signal-to-background ratio for single RNA tracking in the cytoplasm. However, this method has limitations in tracking single RNA in the nucleus and in quantifying RNA expression levels by deep tissue or whole animal imaging that provides relatively low spatial resolution.

Recently, a split FP approach, which was developed to study protein–protein interactions (Kerppola 2006), was adopted to eliminate the background signal in RNA imaging. In the bimolecular fluorescence complementation (BiFC) assay, nonfluorescent FP fragments reconstitute a complete fluorescent protein when they are brought into close proximity. Using two different RBPs conjugated to split FP fragments, several research groups have demonstrated BiFC-based RNA imaging (Rackham and Brown 2004; Ozawa et al. 2007; Valencia-Burton et al. 2007; Yamada et al. 2011; Yiu et al. 2011; Wu et al. 2014). For example, the PP7 system consisting of the PP7 coat protein (PCP) and the PP7 binding site (PBS) can be used in conjunction with the MS2 system for BiFC-based RNA imaging (Wu et al. 2014). In this MS2-PP7 hybrid system, a target RNA was tagged with an alternating tandem array of MS2 and PP7 binding sites (12 × MBS–PBS). MCP and PCP were fused with the N- and C-fragments of the yellow fluorescent protein Venus, respectively. Because the split Venus fragments form a complete FP when the MCP and PCP are bound to the 12 × MBS–PBS-tagged RNA, this approach enables background-free RNA imaging at a single-molecule level. However, a limitation of the BiFC-based approach is that there is a time delay between the production of the target RNA and the generation of the fluorescence signal. Because the folding and maturation of FPs require a substantial amount of time ranging from several minutes to a few hours, BiFC-based RNA imaging techniques have been considered to be suitable only for imaging long-lived RNAs (Xia et al. 2017). Moreover, the time delay for the BiFC signal hampers the detection of nuclear RNA and nascent RNA being made at transcription sites.

Here, we report a real-time background-free imaging method using split superfolder GFPs (sfGFPs) for a next-generation RNA probe in living cells. Taking advantage of the properties of sfGFPs, such as improved folding kinetics (Pédelacq et al. 2006; Andrews et al. 2007) and a relatively fast maturation rate (Pédelacq et al. 2006; Iizuka et al. 2011; Khmelinskii et al. 2012; Balleza et al. 2018), we have extended the utility of split FP-based single RNA imaging in live cells. Two variants of the split sfGFP, which were developed by directed evolution of two or three nonfluorescent fragments of sfGFP (Cabantous et al. 2005, 2013), are fused to MCP and PCP. The so-called bipartite and tripartite split GFPs fused to capsid proteins can successfully bind to the 12 × MBS–PBS-tagged RNA and thus are brought together to reconstitute a complete mature sfGFP. We report that the tripartite sfGFP is particularly suitable for observing mRNA dynamics not only in the cytoplasm but also in the nucleus at a single-molecule resolution. Our results show that the sfGFP-based fluorescence complementation (FC) method is a powerful tool for genetically encoded RNA imaging, providing opportunities for investigators to observe diverse RNA dynamics with high spatiotemporal resolution.

## RESULTS

### Bipartite sfGFP with the MBS–PBS system

**FIGURE 1.**
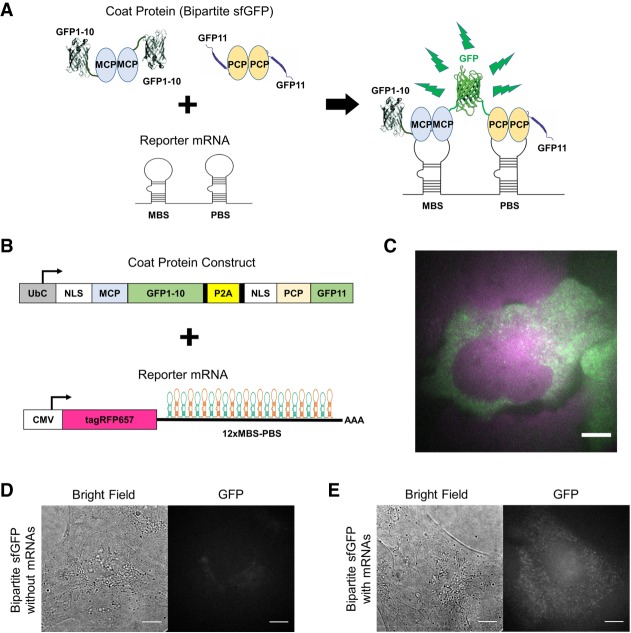
Bipartite sfGFP with MBS–PBS system for mRNA imaging in live cells. (*A*) Schematic of the bipartite sfGFP with the MBS–PBS system in living cells. The two fragments (GFP1–10 and GFP11) are brought together by adjacent binding sites (MBS–PBS) to form a complete sfGFP. (*B*) The MCP and PCP are fused with the bipartite sfGFP fragments (*upper*), and the reporter mRNA expressing tagRFP657 is tagged with 12 × MBS–PBS (*lower*). In the coat protein construct, an NLS was added to the MCP and PCP to accumulate free coat proteins inside the nucleus. (*C*) Fluorescence image of a U2OS cell after lentiviral transfection of the bipartite sfGFP system and the reporter mRNA. The merged image shows the successful reconstitution of the bipartite sfGFP (green) after binding with the reporter mRNA expressing tagRFP657 (magenta). (*D*) Bright-field and fluorescence image of a U2OS cell transfected with only the bipartite sfGFP system. (*E*) Bright-field and fluorescence image of a U2OS cell transfected with both the bipartite sfGFP system and reporter mRNA. Scale bars, 8 µm.

A schematic diagram shows the design of the bipartite sfGFP system for low-background RNA tagging in living cells (Fig. 1A). In this system, coat proteins fused with bipartite sfGFPs bind to their corresponding RNA motifs, restoring a fully fluorescent sfGFP. GFP1–10 and GFP11, which were generated by splitting sfGFP between the 10th and the 11th β-strands (Cabantous et al. 2005), were fused to MCP and PCP, respectively (Fig. 1B; Supplemental Table S1). A tandem array of 12 × MBS–PBS was inserted into the 3′ untranslated region (3′UTR) of the reporter mRNA that encoded tagRFP657 (Fig. 1B; Supplemental Table S2). To increase the coexpression efficiency, MCP–GFP1–10 and PCP–GFP11 were combined into a polycistronic plasmid using a P2A sequence. An NLS peptide was attached to the coat proteins to accumulate the proteins in the nucleus for immediate tagging of nascent mRNAs. The coat protein construct and the reporter mRNA were then coexpressed in U2OS cells using lentiviral transfection. Double-positive cells were sorted by FACS and used for live-cell imaging. The efficient cleavage of the P2A peptide was verified by western blot (Supplemental Fig. S1). Reporter mRNA particles labeled with the bipartite sfGFP were visible in the cytoplasm with little background signal in the nucleus (Fig. 1C) compared to the high background in the nucleus when using the traditional, intact MS2–GFP system (Supplemental Fig. S2). To assess nonspecific signal from the random collision of two split sfGFP fragments, we compared cells expressing the bipartite sfGFP system in the absence and presence of the reporter mRNA (Fig. 1D,E). We observed minimal nonspecific signal in the negative control (Fig. 1D), confirming that the observed GFP signal in Figure 1E was due to complementation of the sfGFP fragments on the reporter mRNAs.

Next, we compared the bipartite sfGFP system with the previously reported bipartite Venus system (Wu et al. 2014). Cells were imaged with the same excitation power (∼132 mW/cm^2^) through the corresponding filter cube for each system. The mRNA intensities were measured from the images by using TrackNTrace software (Stein and Thiart 2016). Overall, the fluorescence intensity of the mRNAs labeled with the bipartite sfGFP system was similar to that of the mRNAs labeled with the split Venus system (Supplemental Fig. S3).

### Tripartite sfGFP with the MBS–PBS system

**FIGURE 2.**
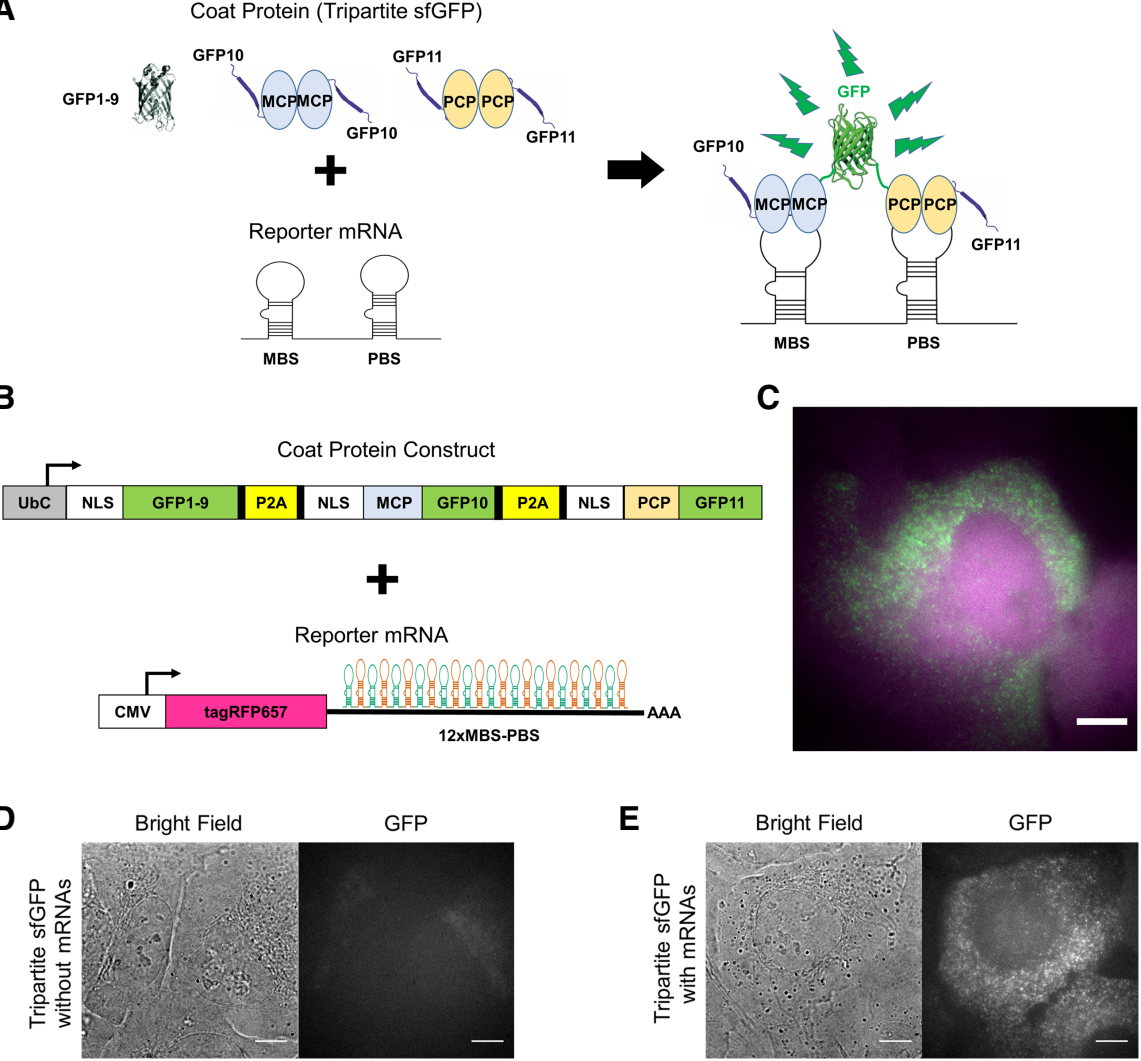
Tripartite sfGFP with MBS–PBS system for mRNA imaging in live cells. (*A*) Schematic of the tripartite sfGFP with the MBS–PBS system in living cells. The small fragments (GFP10 and GFP11) are brought together by adjacent binding sites (MBS–PBS) and combine with the large fragment (GFP1–9) to form a complete sfGFP. (*B*) The MCP and PCP are fused with GFP10 and GFP11, respectively and are coexpressed with GFP1–9 using two P2A sequences (*upper*). The same reporter mRNA used with the bipartite sfGFP system (*lower*) was utilized. (*C*) Fluorescence image of a U2OS cell after lentiviral transfection of the tripartite sfGFP system and the reporter mRNAs. The merged image shows the successful reconstitution of the tripartite sfGFP (green) after binding with the reporter mRNA expressing tagRFP657 (magenta). (*D*) Bright-field and fluorescence image of a U2OS cell transfected with only the tripartite sfGFP system. (*E*) Bright-field and fluorescence image of a U2OS cell transfected with both the tripartite sfGFP system and reporter RNA. Scale bars, 8 µm.

We then adopted a tripartite complementation system to improve the signal-to-noise ratio of the single RNA imaging (Fig. 2A). Recently, a tripartite sfGFP system was developed to enhance the rate of fluorescence generation and to reduce the self-assembly background by using multiple rounds of directed evolution (Cabantous et al. 2013). This tripartite sfGFP system consists of two small fragments, GFP10 (residues 194–212) and GFP11 (residues 213–233), and a large GFP1-9 fragment (residues 1–193) (Cabantous et al. 2013). GFP10 and GFP11 were fused to MCP and PCP, respectively. GFP1–9, MCP–GFP10, and PCP–GFP11 were combined in a single vector using two P2A sequences (Fig. 2B; Supplemental Table S3), and were coexpressed along with the reporter mRNA in U2OS cells (Fig. 2C). Again, the coat protein construct alone did not generate GFP signal (Fig. 2D). Only when both the coat protein construct and the reporter mRNA were coexpressed did we observe diffraction-limited spots with strong GFP signal in the nucleus and cytoplasm (Fig. 2E). This result indicates the efficient combination of all three fragments (GFP10, GFP11, and GFP1–9) only in the presence of the reporter mRNA.

To test whether the tripartite sfGFP system hinders the degradation of the reporter mRNA, we inhibited transcription with 100 µM 5,6-dichloro-1-β-d-ribofuranosylbenzimidazole (DRB) and imaged the same cells at different time points (Supplemental Fig. S4A). The number of sfGFP-tagged mRNAs per cell significantly decreased at 1.5 and 5.5 h after the treatment (Supplemental Fig. S4B, *P* < 0.05; Student's *t*-test). We did not observe any noticeable accumulation of tagged mRNA decay fragments that had been previously reported in yeast (Garcia and Parker 2015, 2016; Heinrich et al. 2017). The average sfGFP background level inside a cell was similar at 0.5, 1.5, and 5.5 h after the treatment (Supplemental Fig. S4C). Although the association of tripartite sfGFP is known to be irreversible (Cabantous et al. 2013), we did not observe a noticeable increase in the background over several passages of the cells after lentiviral transfection.

### Comparison of the bipartite and tripartite sfGFP systems for RNA imaging

**FIGURE 3.**
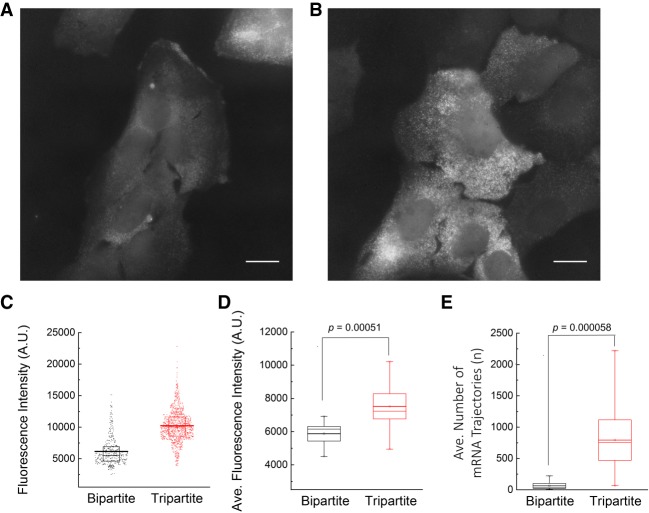
Comparison of the bipartite and tripartite sfGFP systems for single-mRNA imaging. (*A*) Fluorescence image of U2OS cells expressing the reporter mRNA labeled with the bipartite sfGFP system. (*B*) Fluorescence image of U2OS cells expressing the reporter mRNA labeled with the tripartite sfGFP system. Scale bars, 20 µm. (*C*) Fluorescence intensity of single mRNAs detected from a cell transfected with the bipartite (black) or tripartite (red) sfGFP system. Each dot represents the fluorescence intensity of an individual mRNA. The mean value is marked with a horizontal line. (*D*) The average fluorescence intensity of mRNAs labeled with each split sfGFP system (*n* = 17 cells for each system). (*E*) The average number of mRNA trajectories detected by each system (*n* = 17 cells for each system). The mean values are marked with open squares with thick, horizontal lines. The boxplots show the median (horizontal line in the box), first and third quartile (lower and upper hinges of the box, respectively), with whiskers from the minimum to maximum values. The *P*-values were determined by Student's *t*-test.

To compare the brightness of the reporter mRNAs labeled with the bipartite and tripartite sfGFP constructs, we measured the fluorescence amplitude of the particles under the same imaging conditions (Fig. 3A,B). Using TrackNTrace software (Stein and Thiart 2016), we obtained the trajectory and the fluorescence intensity of mRNAs from the time-lapse images taken at a 10 Hz frame rate. Figure 3C shows the overall distribution of the fluorescence intensity of mRNAs detected in a cell transfected with either the bipartite or tripartite system. The mRNAs labeled with the tripartite sfGFP system had a higher mean fluorescence intensity than those labeled with the bipartite sfGFP system. The average fluorescence intensity (Fig. 3D) and the average number of detected mRNA trajectories per cell (Fig. 3E) were also higher in the tripartite than in the bipartite system (*P* < 0.005 by Student's two-tailed *t*-test, *n* = 17 cells for each system). These results indicate that the tripartite sfGFP system provides a higher signal-to-noise ratio than the bipartite system.

To confirm that the detected particles were indeed single mRNA molecules, we performed single-molecule fluorescence in situ hybridization (smFISH) for the reporter mRNA (Supplemental Fig. S5). We designed three smFISH probes targeting the MS2–PP7 stem–loop linker sequences, which had a total of 36 binding sites for a single mRNA (Supplemental Table S4). Both the bipartite (Supplemental Fig. S5A) and tripartite (Supplemental Fig. S5B) sfGFP systems showed good overlap with the smFISH signal. Although fixation and smFISH procedures caused a decrease of GFP fluorescence, we were able to detect single mRNA molecules labeled with both smFISH probes and sfGFPs. The detection efficiencies of the bipartite and tripartite sfGFP systems after smFISH (Supplemental Fig. S5C) were estimated by using a previously described analysis method (Horvathova et al. 2017). As expected, the detection efficiency of the tripartite system (58 ± 4%) was much higher than that of the bipartite system (39 ± 6%). Because the fluorescent intensity of sfGFP is higher in live cells, the actual detection efficiencies of the split systems in live cells would be higher than the values presented in Supplemental Figure S5C.

### Dynamics of single mRNAs labeled with the tripartite sfGFP system

**FIGURE 4.**
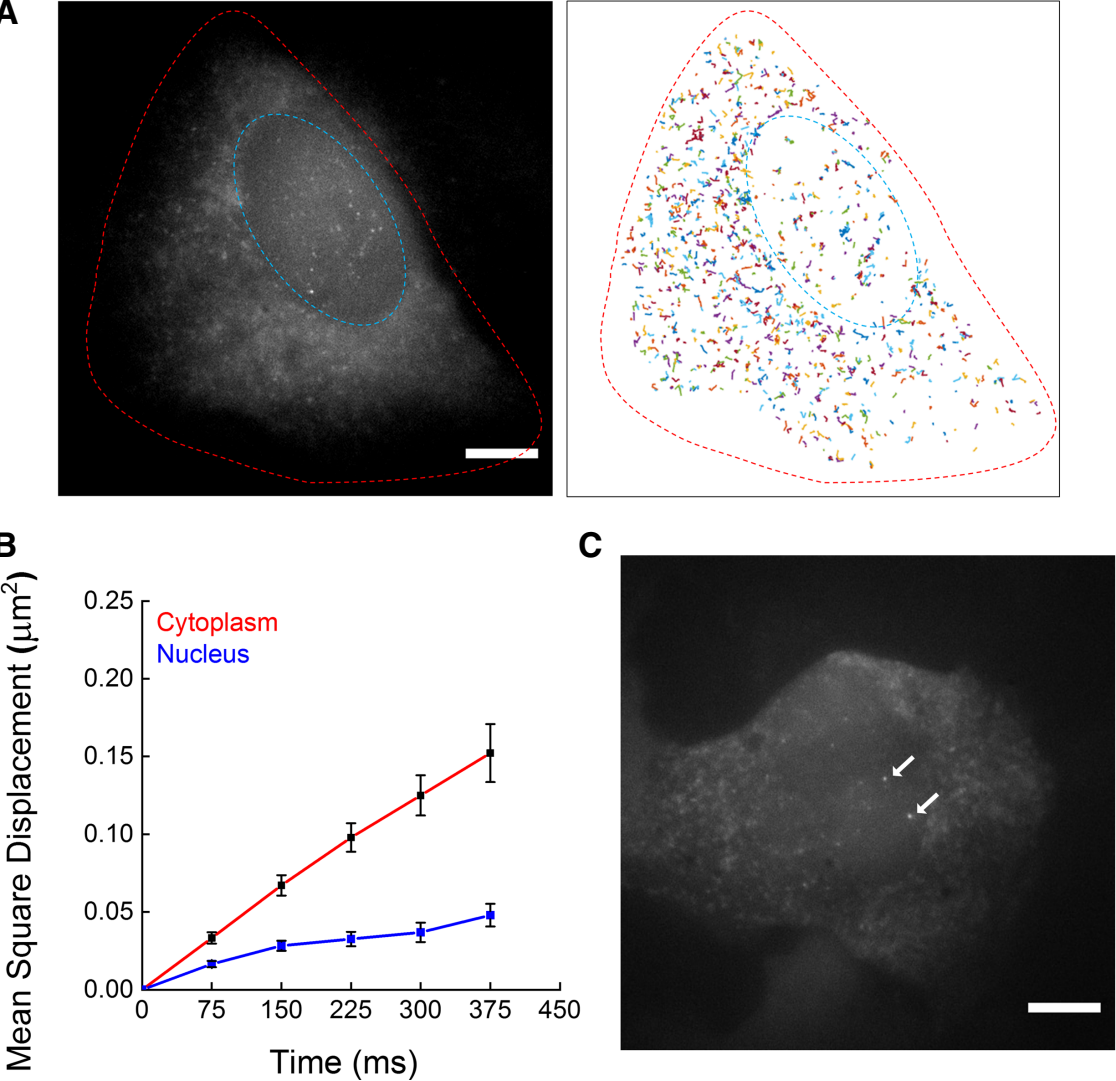
Single mRNA dynamics inside a living cell detected with the tripartite sfGFP system. (*A*) Fluorescence image of single mRNA molecules in a U2OS cell transfected with the tripartite sfGFP system. Nuclear mRNAs (inside the blue dashed line) are clearly visible, as well as mRNAs in the cytoplasm (between the red and blue dashed lines) (*left*). The trajectories of the mRNAs are shown in various colors (*right*). See Supplemental Movie S1 online. (*B*) The EAMSD curves of the mRNAs in the cytoplasm (red line) and the nucleus (blue line). Error bars represent SEM (*n* = 13 cells). (*C*) Two transcription sites detected inside a nucleus (arrows). See Supplemental Movie S2 online. Scale bars, 8 µm.

Previous BiFC-based RNA imaging tools visualized only cytoplasmic mRNAs due to the slow response time of the split system. Because the nuclear export of mRNA occurs within 5–40 min after transcription (Mor et al. 2010), folding and maturation of the split proteins should be completed within this time range to visualize nuclear mRNAs. We found that the tripartite sfGFP system enabled single-molecule imaging of mRNAs in the nucleus (inside the blue dashed line in Fig. 4A), as well as those in the cytoplasm (the area between the red and the blue dashed lines in Fig. 4A). To compare the mobility of mRNA in the nucleus and the cytoplasm (Supplemental Movie S1), we tracked single mRNA particles and plotted their trajectories (Fig. 4A, right). We collected 3629 trajectories in the cytoplasm from 11 cells and 713 trajectories in the nuclei from 13 cells. The ensemble-averaged mean square displacement (EAMSD) of mRNA was calculated using a previously described method (Song et al. 2018). The EAMSD curves of mRNA in the nucleus (blue) and the cytoplasm (red) are plotted in Figure 4B. The diffusion coefficient of mRNA was higher in the cytoplasm (0.10 µm^2^/sec) than in the nucleus (0.02 µm^2^/sec), which was consistent with the result in a previous report (Mor et al. 2010). Furthermore, we were able to observe strong fluorescence signals from 1–2 loci inside the nuclei, which presumably indicate transcription sites (white arrows in Fig. 4C; Supplemental Movie S2). Our results suggest that the tripartite sfGFP with the MBS–PBS system enables background-free imaging of not only cytoplasmic but also nuclear mRNA dynamics.

## DISCUSSION

In this report, we have demonstrated that split sfGFP with the MBS–PBS system is a powerful tool for imaging single mRNA dynamics with minimal background. The overall performance of the bipartite sfGFP system is similar to that of the previously reported split Venus system for imaging single mRNAs (Wu et al. 2014). By adapting the tripartite sfGFP (Cabantous et al. 2013), we have significantly improved the signal-to-background ratio and enabled single mRNA imaging in the nucleus as well as in the cytoplasm in living cells.

Previously, some limitations of BiFC-based RNA imaging have been reported such as (i) background from the spontaneous assembly of split FP, (ii) slow maturation of FPs, and (iii) irreversible association of the FP fragments (Ozawa et al. 2007; Kerppola 2009; Xia et al. 2017). Because spontaneous assembly of three components is much less likely to occur than of two components, the background fluorescence can be further suppressed by using the tripartite sfGFP system. Moreover, the synergistic effect of the relatively short maturation time of sfGFP (Pédelacq et al. 2006; Iizuka et al. 2011; Khmelinskii et al. 2012; Balleza et al. 2018) and the improved folding and complementation efficiency of the tripartite sfGFP system (Cabantous et al. 2013) allowed visualization of transcription sites and single mRNAs in the nucleus. The tripartite sfGFP used in our experiment has a different amino acid sequence from that of the bipartite sfGFP. Therefore, we cannot simply attribute the better performance of the tripartite sfGFP system to the difference between binary and ternary interactions. It is possible that other improved bipartite split FP systems (Huang et al. 2015; Feng et al. 2017; Köker et al. 2018) may perform as well as the tripartite sfGFP system tested in this report.

Because the association of split sfGFP is known to be irreversible (Cabantous et al. 2013), we investigated whether the reconstituted sfGFP hinders mRNA degradation or increases the background fluorescence over time. The number of sfGFP-labeled mRNAs significantly decreased at 1.5 and 5.5 h after inhibition of transcription by DRB treatment. And we did not observe any significant accumulation of sfGFP-tagged mRNA decay fragments. While there have been some concerns about the possible accumulation of MS2-labeled mRNA decay fragments in bacteria (Golding and Cox 2004) and budding yeast (Garcia and Parker 2015, 2016; Haimovich et al. 2016; Heinrich et al. 2017), recent studies have reported that such degradation artifacts are not observed using the traditional MS2–GFP system in mammalian cells and tissues (Horvathova et al. 2017; Tutucci et al. 2018; Kim et al. 2019). Because the average half-life of mRNA in mammalian cells (several hours) is much longer than in bacteria (∼5 min) and yeast (∼23 min), MS2 and PP7 RNA labeling methods may be less prone to the degradation artifact in higher organisms (Das et al. 2018; Tutucci et al. 2018; Kim et al. 2019). In addition, we did not find a significant increase in the background fluorescence after the reporter mRNAs were degraded. The accumulation rate of background fluorescence depends on several factors such as the lifetime and expression level of the tagged mRNA, the lifetime of reconstituted sfGFP, and the cell division rate. We empirically found that the irreversibility of split sfGFP did not hamper single-mRNA imaging in this study.

We anticipate that the FC-based RNA imaging technology will have a great potential for intravital imaging of RNA because of the minimal background noise. An optimal candidate for intravital imaging is a red-shifted split fluorescent protein (Chu et al. 2009; Filonov and Verkhusha 2013; Han et al. 2014; Chen et al. 2015) due to less absorbance and light scattering in tissue. In addition to split fluorescent proteins, there are various two-hybrid systems for in vivo imaging modalities, such as bioluminescence and positron emission tomography (PET) (Shekhawat and Ghosh 2011). For instance, Gambhir and coworkers engineered a red light-emitting bioluminescence resonance energy transfer (BRET) system (Dragulescu-Andrasi et al. 2011) and a PET-based split reporter system using herpes simplex virus type 1 thymidine kinase (HSV1-TK) (Massoud et al. 2010). Any newly developed protein–protein interaction reporters could be combined with the MBS–PBS system for background-free RNA detection using various molecular imaging techniques, providing a powerful tool for revealing the complex dynamics of gene expression in vivo.

## MATERIALS AND METHODS

### Cloning and plasmid construction

All plasmids for the split sfGFPs with the MBS–PBS hybrid system were constructed in lentiviral vectors. To generate the reporter mRNA construct with the 12 × MBS–PBS, we replaced CFP in the phage-CMV-CFP-12 × MBS–PBS plasmid (gift from Dr. Robert H. Singer) with tagRFP657. To generate the polycistronic vectors with the bipartite and tripartite sfGFPs, we amplified nls-ha-MCP and nls-ha-PCP by polymerase chain reaction (PCR) from the ubc-nls-ha-MCP-VenusN-nls-ha-PCP-VenusC plasmid (Addgene plasmid #52985), synthesized the sfGFP fragments, and inserted them into the pCCLsin.PPT.UbiC.GFP lentiviral backbone (Follenzi and Naldini 2002). The amino acid and DNA sequences are provided in Supplemental Tables 1–3.

### Lentivirus production and transfection

Lentiviral vectors for the coat proteins were prepared by cotransfecting 293T cells with the third-generation packaging constructs (pMDLg/pRRE, pRSV-REV, and pMD2.VSVG) and the transfer vector by calcium phosphate precipitation. The culture media was replaced with high-glucose Dulbecco's modified Eagle medium (DMEM, Thermo Fisher Scientific) supplemented with 10% fetal bovine serum (FBS), 1% GlutaMAX (Thermo Fisher Scientific), and 0.25% penicillin-streptomycin (PS, Thermo Fisher Scientific) at 12–16 h after transfection. After 24 and 48 h, the supernatant was collected and filtered with a 0.22 µm syringe filter.

The lentiviral vector for the reporter mRNA was prepared similar to a previously described method (Mostoslavsky et al. 2005). Briefly, the medium was changed to DMEM, 10% FBS, and 1% GlutaMAX (without antibiotics) at least 1 h before transfection. Next, 293T cells were transfected with Gag-Pol, Rev, Tat, VSVG, and the transfer vector by using Fugene HD transfection reagent (Promega). The culture media was replaced with DMEM supplemented with 10% FBS, 1% GlutaMAX, and 1% PS at 12–16 h after transfection. After 36 h, the supernatant was collected and filtered with a 0.22 µm syringe filter. The viral production and titration were confirmed (>5 × 10^5^ IFU/mL) with the Lenti-X GoStix kit (Clontech).

The human osteosarcoma U2OS cell line was purchased from the Korean Cell Line Bank and grown in DMEM with 10% FBS, 1% GlutaMAX, and 1% PS. For lentiviral transfection, the LV pellet was resuspended in DMEM with polybrene (6 µg/mL, Sigma) and added to the U2OS cells seeded in a six-well plate (1 × 10^5^ per well). The infected U2OS cells were sorted with a FACS Aria II (BD Biosciences). The positive cells expressing both GFP and tagRFP657 were collected and used for live-cell imaging.

### Imaging and tracking single mRNAs in live cells

To perform live-cell imaging, we removed the growth medium from the cell cultures and replaced it with imaging media, which was phenol-red free Leibovitz's L-15 medium (Thermo Fisher Scientific) containing 10% FBS, 1% GlutaMAX, and 1% PS. Wide-field fluorescence images were taken using an Olympus IX73 inverted microscope equipped with a U Apochromat 150× 1.45 NA objective (Olympus), two iXon Ultra 897 electron-multiplying charge-coupled device (EMCCD) cameras (Andor), an MS-2000 XYZ automated stage (ASI), and a Chamlide TC top-stage incubator system (Live Cell Instrument). 488 nm and 561 nm diode lasers (Cobolt) were used to excite the GFP and tagRFP657, respectively. The fluorescence emission was filtered with 525/50 and 605/52 bandpass filters (TRF89902-EM, Chroma). Time-lapse images for RNA tracking were taken at 20 frames per second (fps) with a 50 msec exposure time using Micro-Manager software. Tracking of a single mRNA particle was performed with TrackNTrace software (Stein and Thiart 2016). The first six points of the EAMSD curves were fitted to obtain the diffusion coefficients.

For transcription inhibition experiments, cells were imaged after treatment with 100 µM DRB (Sigma D1916). *Z*-section images of cells were taken at 0.5, 1.5, and 5.5 h after DRB treatment. After the maximum projection of *z*-stack images, mRNAs were detected by the TrackNTrace software (Stein and Thiart 2016). The background level was determined by obtaining the median pixel intensity in the cytoplasm.

To compare the previously reported split Venus-based reporter system (Wu et al. 2014) and our bipartite system, all of the images were taken in the same imaging conditions. As Venus and GFP have different fluorescence spectra, we measured the fluorescence intensity of the RNA particles under the same LED power density at 132 ± 2 mW/cm^2^ measured with the corresponding excitation filter. The mRNAs were again detected by automated particle detecting software (Stein and Thiart 2016) and analyzed.

### Western blot

Proteins (14 µg) obtained from lentivirus-infected cell lines were separated on 4%–12% Bis-Tris polyacrylamide precast gels in MES-SDS running buffer in a reducing condition and transferred to nitrocellulose membranes by a Mini Blot Module (Thermo Scientific). Anti-GFP (1:1000, A6455, Thermo Scientific) and anti-GAPDH (1:20000 G9545, Sigma) were used as primary antibodies, and anti-rabbit IgG conjugated to HRP (1:5000, SA002-500, GenDEPOT) was used as secondary antibody. Pierce ECL western blotting substrate (Thermo Scientific) was used for HRP detection. The western blots were imaged by a LAS 4000 (GE Healthcare Life Sciences).

### Single-molecule fluorescence in situ hybridization (smFISH) and colocalization analysis

Cells were fixed with 4% paraformaldehyde (PFA) in phosphate-buffered saline (PBS). After permeabilization with 0.1% Triton X-100 in PBS for 10 min at room temperature (RT), the cells were prehybridized with 10% formamide in 2× SSC for 30 min at RT. Hybridization was performed at 37°C for 3 h using hybridization buffer (0.1 µM 20-mer DNA probes [Supplemental Table 4], 2× SSC, 10% formamide, 10% dextran sulfate, 2 mg/mL bovine serum albumin [BSA], 0.025 mg/mL *Escherichia coli* transfer RNA, and 0.025 mg/mL sheared salmon sperm DNA in ribonuclease [RNase]-free water). The cells were then washed twice with warm 10% formamide in 2× SSC for 20 min, followed by multiple washings with 2× SSC and DAPI staining. For colocalization analysis, the cells were imaged in PBS using an Olympus IX73 inverted microscope equipped with a U Apochromat 150× 1.45 NA objective (Olympus), an iXon Ultra 897 EMCCD camera (Andor), a SOLA SE light-emitting diode (Lumencor), an EGFP filter set (Chroma, 49002) and a Cy3/TRITC filter set (Chroma, 49004). After registration of the two-color images, particles were detected with the TrackNTrace software (Stein and Thiart 2016). If the distance between two particles in two different channels were shorter than 300 nm, it was counted as colocalization. The detection efficiencies of the split systems were calculated by using the method described by Horvathova et al. (2017).

## SUPPLEMENTAL MATERIAL

Supplemental material is available for this article.

## Supplementary Material

Supplemental Material
