# Supplementary material for: Live-cell imaging of single mRNA dynamics using split superfolder green fluorescent proteins with minimal background
Source: RNA. 2020 Jan;26(1):101–9. doi: 10.1261/rna.067835.118 (PMC6913125; doi:10.1261/rna.067835.118)
Supplement: Supplemental Material [file supp_26_1_101__index.html]

Live-cell imaging of single mRNA dynamics using split superfolder green fluorescent proteins with minimal background — Live-cell imaging of single mRNA dynamics using split superfolder green fluorescent proteins with minimal background — Supplemental Material 

# Live-cell imaging of single mRNA dynamics using split superfolder green fluorescent proteins with minimal background

## Supplemental Material

- Supplemental\_Movie\_S1.avi
- Supplemental\_Movie\_S2.avi
- Supplemental\_Text.pdf
