# Supplementary material for: Live-cell imaging of single mRNA dynamics using split superfolder green fluorescent proteins with minimal background
Source: RNA. 2020 Jan;26(1):101–9. doi: 10.1261/rna.067835.118 (PMC6913125; doi:10.1261/rna.067835.118)
Supplement: Supplemental Material [file supp_067835.118_Supplemental_Text.pdf]

Supplemental Material for

Live-cell imaging of single mRNA dynamics using split superfolder green fluorescent proteins with minimal background

Sung Young Park[1,2], Hyungseok C. Moon[1] and Hye Yoon Park[1,3,4]*

[1] Department of Physics and Astronomy, Seoul National University, Seoul, 08826, Korea

[2] Center for RNA Research, Institute for Basic Science, Seoul, 08826, Korea

[3] Institute of Applied Physics, Seoul National University, Seoul, 08826, Korea

[4] Institute of Molecular Biology and Genetics, Seoul National University, Seoul, 08826, Korea

*Correspondence: hyeyoon.park@snu.ac.kr

This file includes:

Supplemental Figures S1-S5

Supplemental Tables S1-S4

Captions for Supplemental Movies S1 and S2

Other supplemental material for this manuscript includes the following:

Supplemental Movies S1 and S2

Supplemental Figures

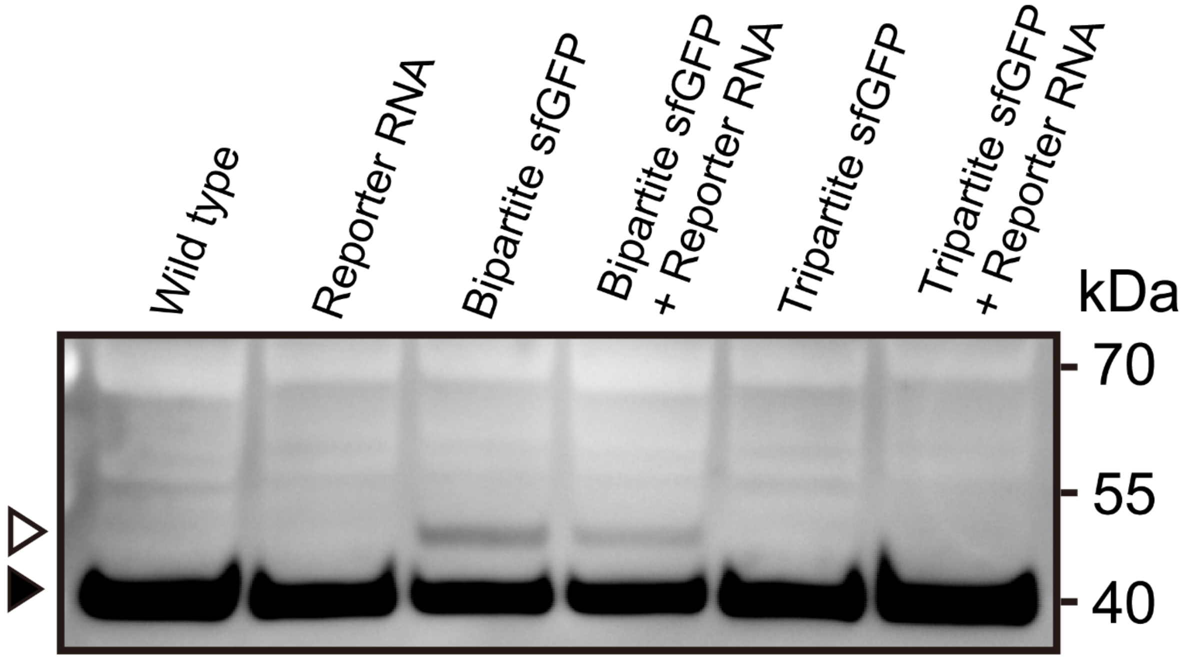

Figure S1. Western blot for the bipartite and tripartite sfGFP-fused coat protein constructs containing P2A sequences. The MCP-GFP1-10 fusion protein (▷ bands, ~43 kDa) can be observed with anti-GFP antibody in the cell lines expressing the bipartite sfGFP construct. Other cell lines show only the GAPDH loading control (▶ bands, ~36 kDa). This result indicates that the P2A peptides are properly cleaved to separate the split sfGFP-fused proteins without much residual leakage.

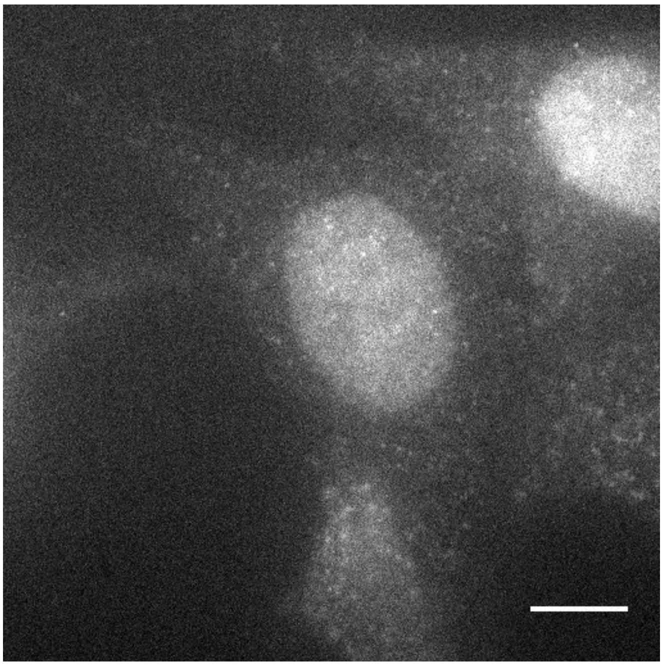

Figure S2. Intact MS2-GFP system with high background signal in the nuclei of MEFs.

Strong background signal appears in the nuclei of mouse embryonic fibroblasts (MEFs) when using the traditional, intact MS2-GFP system. Scale bar, 8 µm.

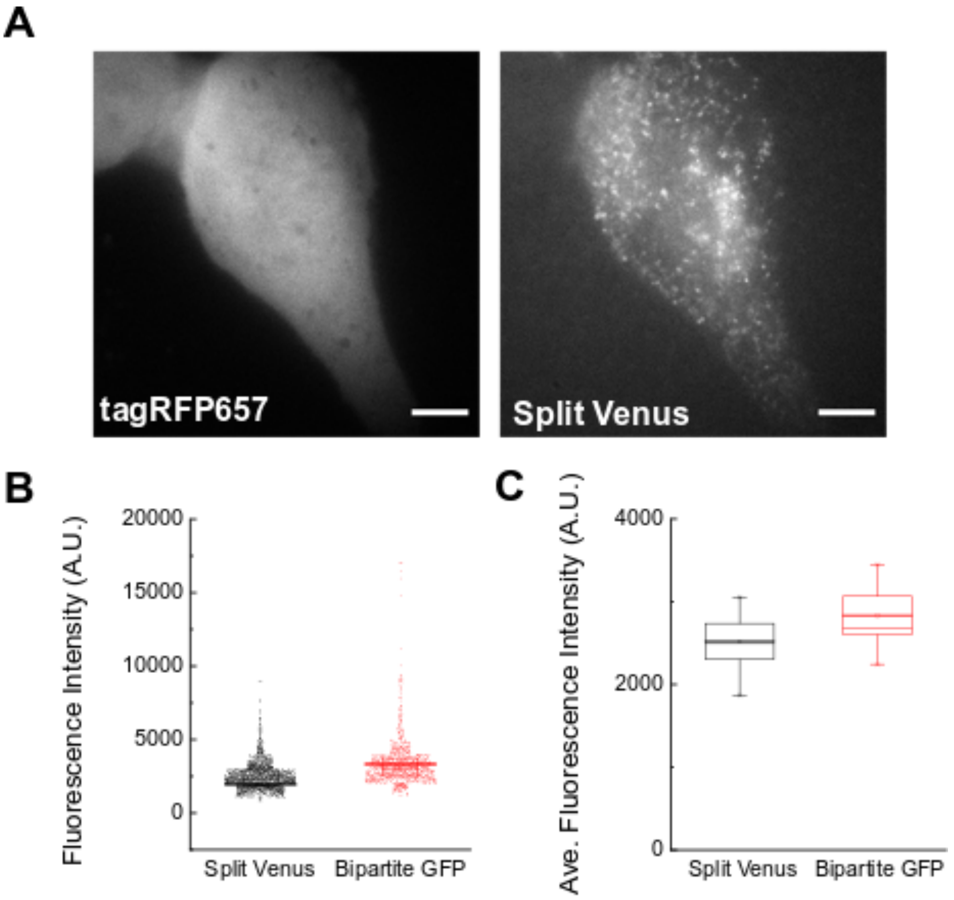

Figure S3. Comparison of the split Venus and bipartite sfGFP systems for RNA imaging. (A) A U2OS cell transfected with the MBS-PBS tagged reporter mRNA expressing tagRFP657 and coat proteins fused with split Venus. Scale bars, 8 µm. **(B)** Fluorescence intensity of single mRNAs detected from a representative cell transfected with the split Venus (black) or bipartite sfGFP (red) system. The mean value is marked with a horizontal line. **(C)** The average fluorescence intensity of the mRNAs labeled with each split system (n = 7 cells for each system).

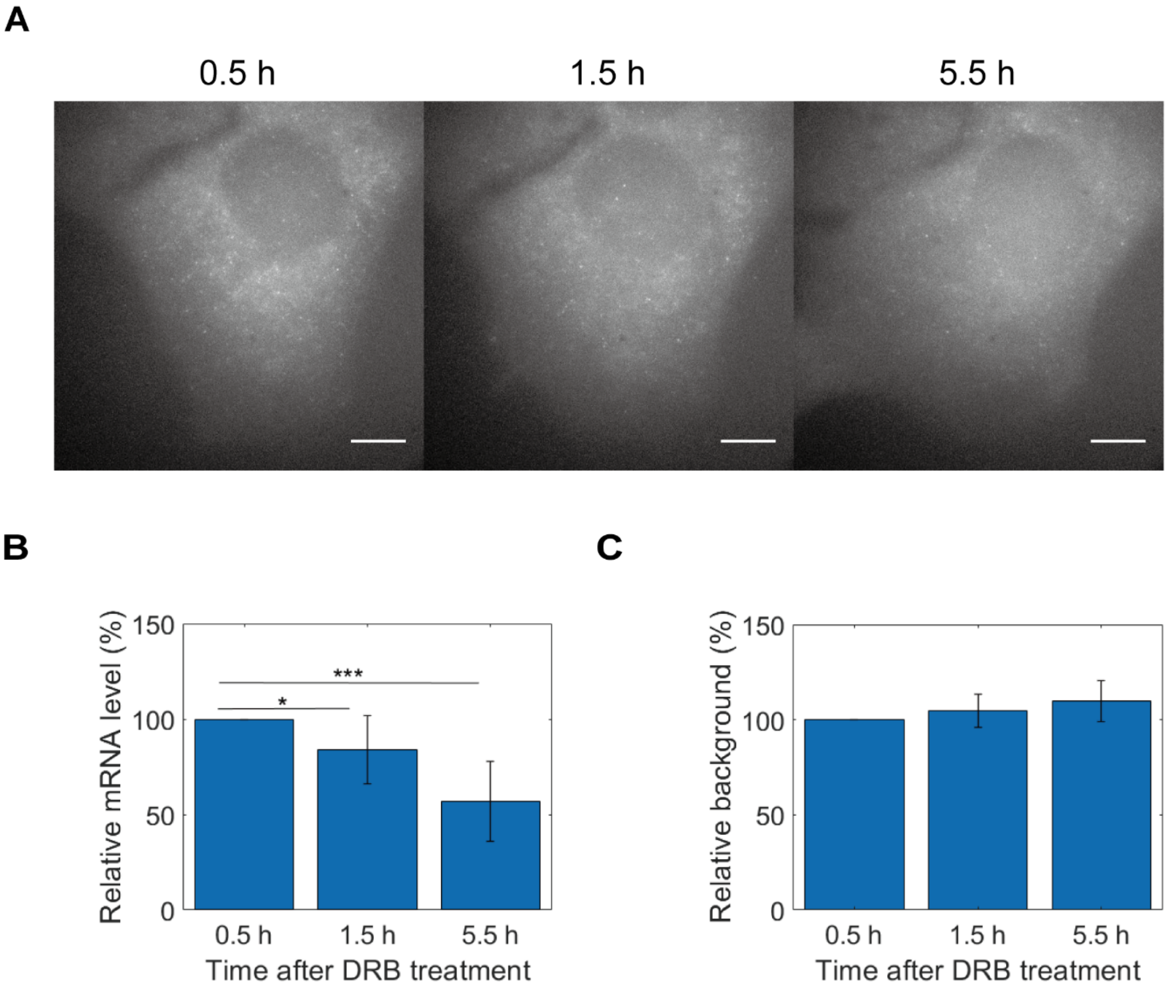

Figure S4. Tripartite sfGFP-tagged reporter mRNA and background level after transcription inhibition. (A) Time-lapse images of a cell after transcription inhibition by 100 µM 5,6-dichloro-1-β-D-ribofuranosylbenzimidazole (DRB) treatment. Scale bars, 8 µm. **(B)** The average number of mRNA per cell normalized to 0.5 h data (***, $p < 0.001$; *, $p < 0.05$; Student's two-tailed t-test). Error bars represent standard deviation (n = 12 cells). **(C)** The average cytoplasmic background level was normalized to 0.5 h data. Error bars represent standard deviation (n = 8 cells).

A

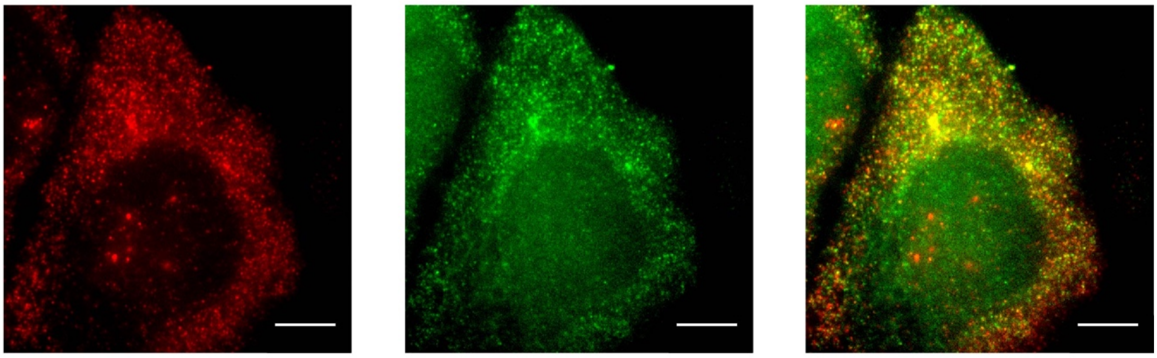

B

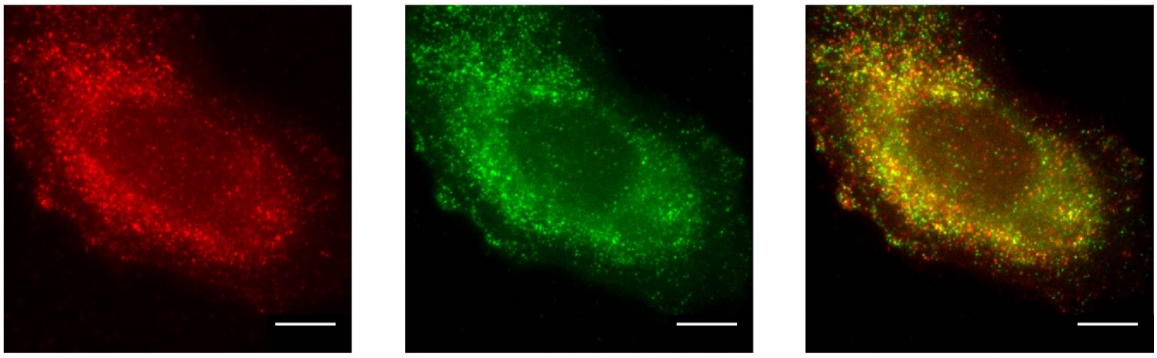

C

Detection efficiencies (%)		
	Bipartite	Tripartite
Nucleus	26 ± 4	46 ± 8
Cytoplasm	45 ± 7	60 ± 2
Whole cell	39 ± 6	58 ± 4

Figure S5. Single-molecule RNA FISH for assessing the bipartite and tripartite sfGFP systems. (A) Fluorescence images of smFISH probes hybridized to the reporter mRNA (left, red) and the bipartite sfGFP system (middle, green) in a U2OS cell. The merged image (right) shows colocalization between the smFISH and the bipartite sfGFP signals. **(B)** Fluorescence images of smFISH probes hybridized to the reporter mRNA (left, red) and the tripartite sfGFP system (middle, green) in a U2OS cell. The merged image (right) shows colocalization between the smFISH and the tripartite sfGFP signals. **(C)** Detection efficiencies of the bipartite and tripartite sfGFP systems. Errors represent standard deviation (n = 5 cells). Scale bars, 8 µm.

Supplemental Tables

Name	Amino acid sequence
NLS-HA-MCP	MGPKKKRKVGYPYDVPDYAIEGRHMLAVKMASNFTQFVLVD<br>NGGTGDVTVAPSNFANGIAEWISSNSRSQAYKVTCSVRQSSAQ<br>NRKYTIKVEVPKGAWRSYLNMELTIPIFATNSDCELIVKAMQGL<br>LKDGNPIPSAIAANSGIYAD
GFP1-10	MSKGEELFTGVVPILVELDGDVNGHKFSVRGEGEGDATIGKLT<br>LKFICTTGKLPVPWPTLVTTLTYGVQCFSRYPDHMKRHDFFKS<br>AMPEGYVQERTISFKDDGKYKTRAVVKFEGDTLVNRIELKGTD<br>FKEDGNILGHKLEYNFNSHNVYITADKQKNGIKANFTVRHNVE<br>DGSVQLADHYQQNTPIGDGPVLLPDNHYLSTQTVLSKDPNEK
P2A	ATNFSLLKQAGDVEENPGPA
NLS-HA-PCP	MGPKKKRKVGYPYDVPDYAIEGRHLASKTIVLSVGEATRTLTEI<br>QSTADRQIFEEKVGPLVGRLRLTASLRQNGAKTAYRVNLKLDQ<br>ADVVDSGLPKVRYTQVWSHDVTIVANSTEASRKSLYDLTKSLV<br>ATSQVEDLVVNLVPLGR
GFP11	RDHMVLHEYVNAAGITA

Table S1. Amino acid sequence of the bipartite sfGFP-coat protein construct used in this study.

Name	DNA sequence
tagRFP657	ATGAGCGAGCTGATCACCGAGAACATGCACATGAAGCTGTACATGGAGGGCACCGTGAACAACCACCACTTCAAGTGCACATCCGAGGGCGAAGGCAAGCCCTACGAGGGCACCCAGACCCAGAGAATCAAGGTGGTCGAGGGCGGCCCTCTCCCCTTCGCCTTCGACATCCTGGCTACCAGCTTCATGTACGGCAGTCACACCTTCATCAACCACACCCAGGGCATCCCCGACTTCTGGAAGCAGTCCTTCCCTGAGGGCTTCACATGGGAGAGAGTCACCACATACGAAGACGGGGGCGTGCTGACCGCTACCCAGGACACCAGCCTCCAGGACGGCTGCCTCATCTACAACGTCAAGATCAGAGGGGTGAACTTCCCATCCAACGGCCCTGTGATGCAGAAGAAAACACTCGGCTGGGAGGCCCACACCGAGATGCTGTACCCCGCTGACGGCGGCCTGGAAGGCAGAACCGCGCTGGCCCTGAAGCTCGTGGGCGGGGGCCACCTGATCTGCAACTTCAAGACCACATACAGATCCAAGAAACCCGCTAAGAACCTCAAGATGCCCGGCGTCTACTATGTGGACTACAGACTGGAAAGAATCAAGGAGGCCGACAAAGAGACCTACGTCGAGCAGCACGAGGTGGCTGTGGCCAGATACTGCGACCTCCCTAGCAAACTGGGGCACAAGCTTAATTAA
MBS	GCACGAGCATCAGCCGTGC
PBS	CGAGCAGACGATATGGCGTCGCTCG

Table S2. DNA sequence of the reporter mRNA construct used in this study.

Name	Amino acid sequence
NLS-HA	MGPKKKRKVGYPYDVPDYAIEGRH
GFP1-9	MRKGEELFTGVVPILIELDGDVNGHKFFVRGEGEGDATIGKLSL KFICTTGKLPVPWPTLVTTLTYGVQCFSRYPDHMKRHDFFKSA MPEGYVQERTIYFKDDGTYKTRAEVKFEGDTLVNRIELKGIDF KEDGNILGHKLEYNFNSHKVYITADKQNNGIKANFTIRHNVED GSVQLADHYQQNTPIGDGPVLLP*
P2A	ATNFSLLKQAGDVEENPGPA
NLS-HA-MCP	MGPKKKRKVGYPYDVPDYAIEGRHMLAVKMASNFTQFVLVD NGGTGDVTVAPSNFANGIAEWISSNSRSQAYKVTCSVRQSSAQ NRKYTIKVEVPKGAWRSYLNMELTIPIFATNSDCELIVKAMQGL LKDGNPIPSAIAANSGIYAD
GFP10	MDLPDDHYLSTQTILSKDL
NLS-HA-PCP	MGPKKKRKVGYPYDVPDYAIEGRHLASKTIVLSVGEATRTLTEI QSTADRQIFEEKVGPLVGRLRLTASLRQNGAKTAYRVNLKLDQ ADVVDSGLPKVRYTQVWSHDVTIVANSTEASRKSLYDLTKSLV ATSQVEDLVVNLVPLGR
GFP11	SEKRDHMVLLEYVTAAGITDAS*

Table S3. Amino acid sequence of the tripartite sfGFP-coat protein construct used in this study.

Name	DNA sequence
MBS-PBS linker 1	GATGAACCCTGGAATACTGG
MBS-PBS linker 2	CTTGGCAATAAGTACCGTAG
MBS-PBS linker 3	TTTGAAGATTCGACCTGGAG

Table S4. Sequence of the probes used for smFISH of the reporter mRNA.

Probes are modified at both 5′ and 3′ ends for conjugation to Quasar 570 (Biosearch).

Captions for Supplemental Movies

Movie S1. Movement of sfGFP-tagged mRNA in a U2OS cell.

Movie of tripartite sfGFP-tagged mRNAs in a U2OS cell shown in Fig. 4A. Time-lapse images were acquired at 20 frames per second (fps) with 50 ms exposure time for 5 seconds. The movie is played at real time speed.

Movie S2. Movie of sfGFP-tagged mRNA in a U2OS cell with transcription sites.

Movie of tripartite sfGFP-tagged mRNAs in a U2OS cell with transcription sites shown in Fig. 4C. Time-lapse images were acquired at 10 fps with 100 ms exposure time for 10 seconds. The movie is played at real time speed.
